# Multi-model radiomics and machine learning for differentiating lipid-poor adrenal adenomas from metastases using automatic segmentation

**DOI:** 10.3389/fonc.2025.1619341

**Published:** 2025-07-18

**Authors:** Shengnan Yin, Ning Ding, Shaocai Wang, Mengjuan Li, Yichi Zhang, Jiacheng Shen, Haitao Hu, Yiding Ji, Long Jin

**Affiliations:** Department of Radiology, Suzhou Ninth Hospital Affiliated to Soochow University: Suzhou Ninth People’s Hospital, Suzhou, China

**Keywords:** adrenal masses, radiomics, contrast-enhanced CT, machine learning, SHAP analysis

## Abstract

**Background:**

Radiomics based on automatic segmentation of CT images has emerged as a highly promising approach for differentiating adrenal adenomas from metastases in clinical practice; however, its preoperative diagnostic value has not been fully evaluated in previously developed methodologies.

**Objective:**

To fully elucidate the diagnostic value of radiomics based on automatic segmentation techniques in differentiating adrenal adenomas from metastases through a retrospective analysis of clinical and contrast-enhanced CT (CECT) data.

**Methods:**

A retrospective analysis was conducted on the clinical and imaging data of 416 patients with adrenal masses larger than 10 mm, who had clinically indicated contrast-enhanced CT (CECT) examinations at our hospital between January 2020 and June 2024. Adrenal lesions were segmented automatically using 3D Slicer, and radiomic features were extracted from the segmented arterial and venous phase images using PyRadiomics. Feature selection and dimensionality reduction were performed using mutual information (MI), minimum redundancy maximum relevance (MRMR), LASSO, and Pearson correlation analysis. Clinical and imaging features were then incorporated into an XGBoost machine learning model, and model performance was evaluated using Area Under Curve (AUC), accuracy, precision, sensitivity, specificity, and F1 score. SHAP analysis was used to interpret the model’s predictions and identify the most influential features.

**Results:**

This study included 221 adenomas and 195 metastases. Significant differences were observed between the two groups in terms of age, lesion size, and contrast washout rate (P < 0.001). After feature extraction, selection, and dimensionality reduction, 15 arterial phase features, 6 venous phase features, and 18 combined features were used for model training. The AUC values of the XGBoost model for the arterial phase, venous phase, combined arterial and venous phase data, and combined arterial, venous phase, and clinical indicators were 0.81, 0.81, 0.88, and 0.92, respectively. Five-fold cross-validation showed that the average scores of XGBoost were 0.868, 0.823, 0.897, and 0.89, respectively. SHAP summary plot for each sample under different features were used to illustrate the interpretability of the model.

**Conclusion:**

A machine learning model, combining multimodal CT radiomics and automatic segmentation technology, enables machine-based clinical features extraction, improves the differentiation between adrenal adenomas and metastases, and provides a reliable foundation for accurate diagnosis and treatment planning.

## 1 Introduction

The incidence of adrenal incidentalomas (Adrenal incidentaloma, AI) is approximately 6.4% (1, 2). Since they are often asymptomatic and discovered incidentally during imaging examinations, they are frequently overlooked (3), making it very challenging for the adrenal incidentalomas diagnosis. More than half of these are adrenal adenomas, which are the most common benign tumors of the adrenal gland (4). On the other hand, the most common malignant adrenal tumors are metastases (5), which typically require early clinical intervention. However, even among patients with a known history of primary malignancy, only 26-36% of adrenal masses are metastatic (6). Therefore, in clinical practice, accurately distinguishing benign adenomas from malignant metastases is crucial for selecting appropriate treatment plans and predicting patient outcomes. The invasiveness and postoperative complications associated with surgical resection or biopsy to determine the nature of the tumor cannot be ignored (7). Typical adrenal adenomas contain lipids, and traditional imaging techniques can detect them through methods such as CT values, relative chemical shift MRI (Chemical-shift MRI, CSI), and rapid washout characteristics of the lesions (8–10), which are of significant reference value in differentiating adrenal adenomas from non-adenomas. However, 30% of adenomas have insufficient fat content, making the differential diagnosis challenging (11). Therefore, in clinical practice, fully analyzing imaging information is of great significance to accurately distinguish atypical adrenal adenomas from metastatic adrenal lesions (12).

In recent years, radiomics has been developed as an emerging quantitative analysis technique, providing new insights into tumor diagnosis and prognosis by extracting and analyzing many features from images (13–15). Combined with advanced automatic segmentation techniques, radiomics can accurately identify and analyze the subtle characteristics of adrenal lesions, thereby improving diagnostic accuracy. More specifically, automatic segmentation techniques can quickly and accurately extract adrenal lesion areas in contrast-enhanced CT images, providing a high-quality data base for subsequent radiomics analysis (16–18). To solve current challenges in CT image analysis and diagnosis, this study aims to explore radiomics based on automatic segmentation techniques in improving the predicting outcomes between adrenal adenomas and metastases. By analyzing many quantitative imaging features and establishing effective predictive models, our analytical method and machine learning model provide a more reliable basis for clinical diagnosis, significantly reduce unnecessary invasive examinations, and improve patient treatment outcomes and quality of life.

## 2 Materials and methods

### 2.1 Patients

We retrospectively analyzed the clinical data of 416 patients who had clinically indicated enhanced CT (CECT) examination in our hospital between January 2020 and June 2024 and showed the presence of adrenal masses greater than 10mm in diameter. Inclusion Criteria: The adrenal lesions included 221 adenomas and 195 metastases. Lesions diagnosed as adrenal adenomas must meet the following criteria: (1) The lesion has a regular shape and smooth margins; (2) The patient has no history of malignancy, and both imaging and clinical diagnoses are consistent with adenoma; (3) The size of the adrenal nodule remains unchanged on follow-up imaging for at least 6 months without any intervention. Lesions diagnosed as adrenal metastases must meet the following criteria: (1) The patient has a known history of malignancy, and both imaging and clinical diagnoses are consistent with adrenal metastasis; (2) Follow-up imaging shows treatment-related changes in the size of the lesion within 3–6 months. Exclusion Criteria: (1) Patients with incomplete CECT protocols; (2) Patients with incomplete reference standard data; (3) Patients with adrenal lesions smaller than 10 mm; (4) Patients with follow-up duration less than 6 months.

These inclusion criteria ensured that the study subjects were adrenal lesions suitable for comparative analysis and enabled an effective evaluation of the application value of contrast-enhanced CT and radiomics machine learning in differentiating adenomas from metastatic tumors.

This retrospective study adhered to the ethical guidelines of the 1975 Declaration of Helsinki and received approval from the Institutional Review Board of Suzhou Ninth People’s Hospital. In addition, written informed consent for publication was obtained from the patient.

### 2.2 Protocol for abdominal CT

A second-generation dual-source multi-detector computed tomography (MDCT) scanner, the SOMATOM Definition Flash (Siemens, Erlangen, Germany), was utilized for the CT scans. The settings included a tube voltage of 120 kVp, and a reference tube current was set at 300 mAs. A scanning thickness and interval were both at 5 mm. The image reconstruction parameters were 1 mm for both thickness and interval. Other parameters comprised a pitch of 0.8, a tube rotation speed of 0.5 s per rotation, and a detector width of 128 mm × 0.6 mm.

Prior to the CT scan, the patient was required to fast for a minimum of 4 hours and engaged in inspiratory training. For the scan, the patient lay in a supine position with hands supporting the head. The process began with acquiring a localization image and conducting a non-contrast CT scan. Subsequently, 80 mL of the non-ionic contrast agent ioversol (HENGRUI MEDICAL HR, China) was administered intravenously via the antecubital vein at a flow rate of 3.5 mL/s using a high-pressure injector (ulrich GmbH & Co.KG), immediately followed by a 20-mL flush of normal saline at the same rate to clear the syringe. Triphasic CT imaging, encompassing arterial, portal venous, and delayed phases, was initiated at 28–32 seconds, 55–65 seconds, and 3–4 minutes post-contrast injection, respectively. The scan covered an area extending approximately 1 cm above the diaphragm to the lower edge of the liver. During the contrast-enhanced portion, a contrast agent tracking method was employed to precisely time the arterial phase, with subsequent acquisitions of the arterial, portal venous, and delayed phase images.

### 2.3 Automatic segmentation

**Figure 1:**
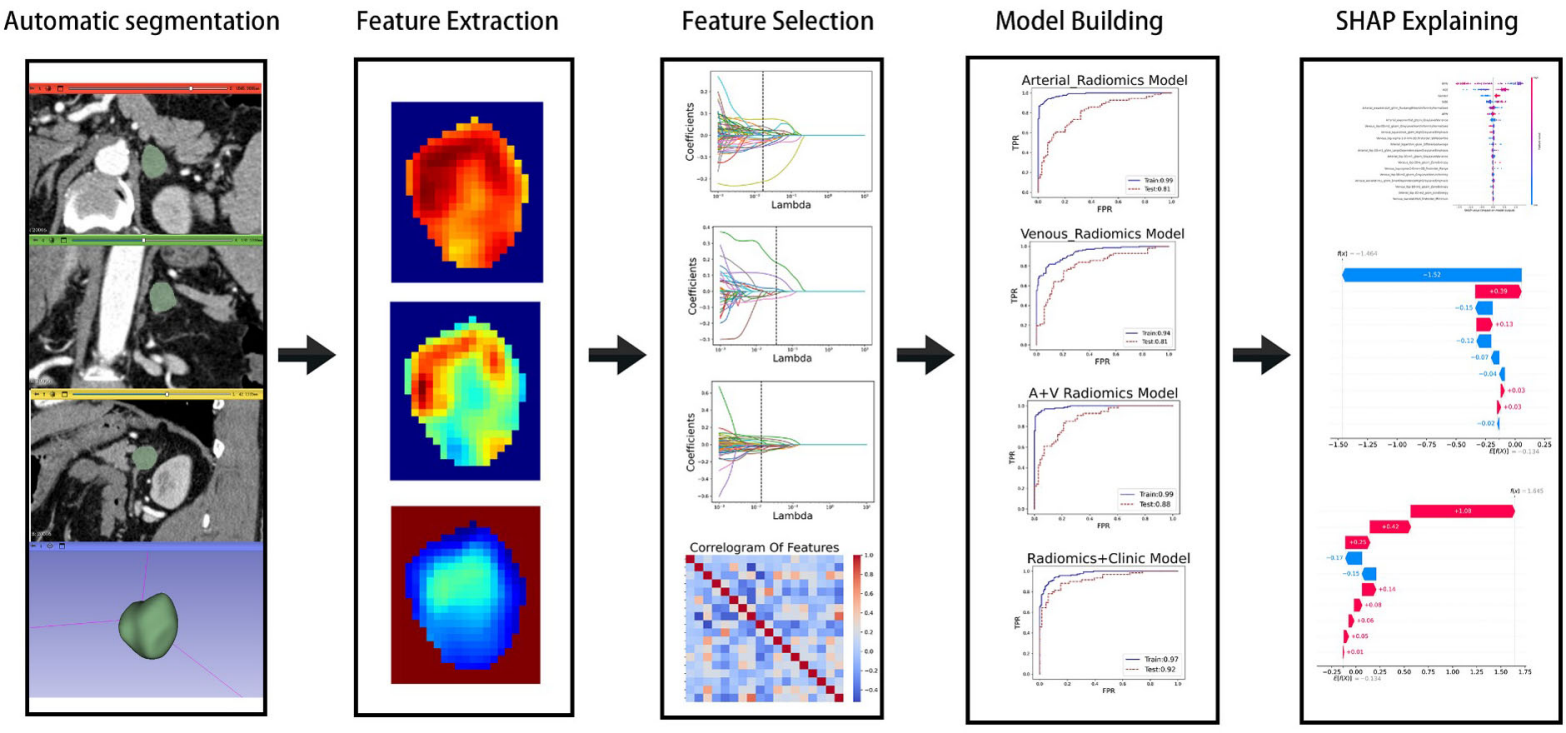
Experimental flow chart.

Thin-section contrast-enhanced CT images contain lesion details. Therefore, we exported arterial and portal venous phase enhanced CT images in DICOM format from the Picture Archiving and Communication System (PACS). After preprocessing and normalization of the input images, the CT image data was imported into the 3D-Slicer plugin (version 5.6.2, [https://www.slicer.org]). A radiologist with ten years of experience attached the ROI and adjusted the size of the rectangular box to encompass the lesion (**Supplementary Figure S1**). The images were then imported into the large model medsam_lite.pth for computation. For patients with inaccurate automatic segmentation results, manual adjustments were made in consultation with experienced senior attending physicians. Finally, the segmentation results were saved. The experimental process is shown in **Figure 1**.

### 2.4 Feature extraction

PyRadiomics (https://www.radiomics.io/pyradiomics.html) was utilized to extract image features from the previously saved segmentation results. In our study, a total of 1,834 imaging features were extracted from both the arterial and venous phases, categorized into seven classes: 1) shape features; 2) first-order statistical features; 3) Gray-Level Co-occurrence Matrix (GLCM) features; 4) Gray-Level Run Length Matrix (GLRLM) features; 5) Gray-Level Size Zone Matrix (GLSZM) features; 6) Gray-Level Dependence Matrix (GLDM) features; and 7) Neighboring Gray-Tone Difference Matrix (NGTDM) features. Additionally, various filters such as Laplacian of Gaussian (LoG, with sigma values of 1.0, 2.0, and 3.0), Wavelet, 3D Local Binary Patterns (LBP3D), Exponential, Square, SquareRoot, Logarithm, and Gradient were applied for feature processing.

### 2.5 Feature selection

**Figure 2:**
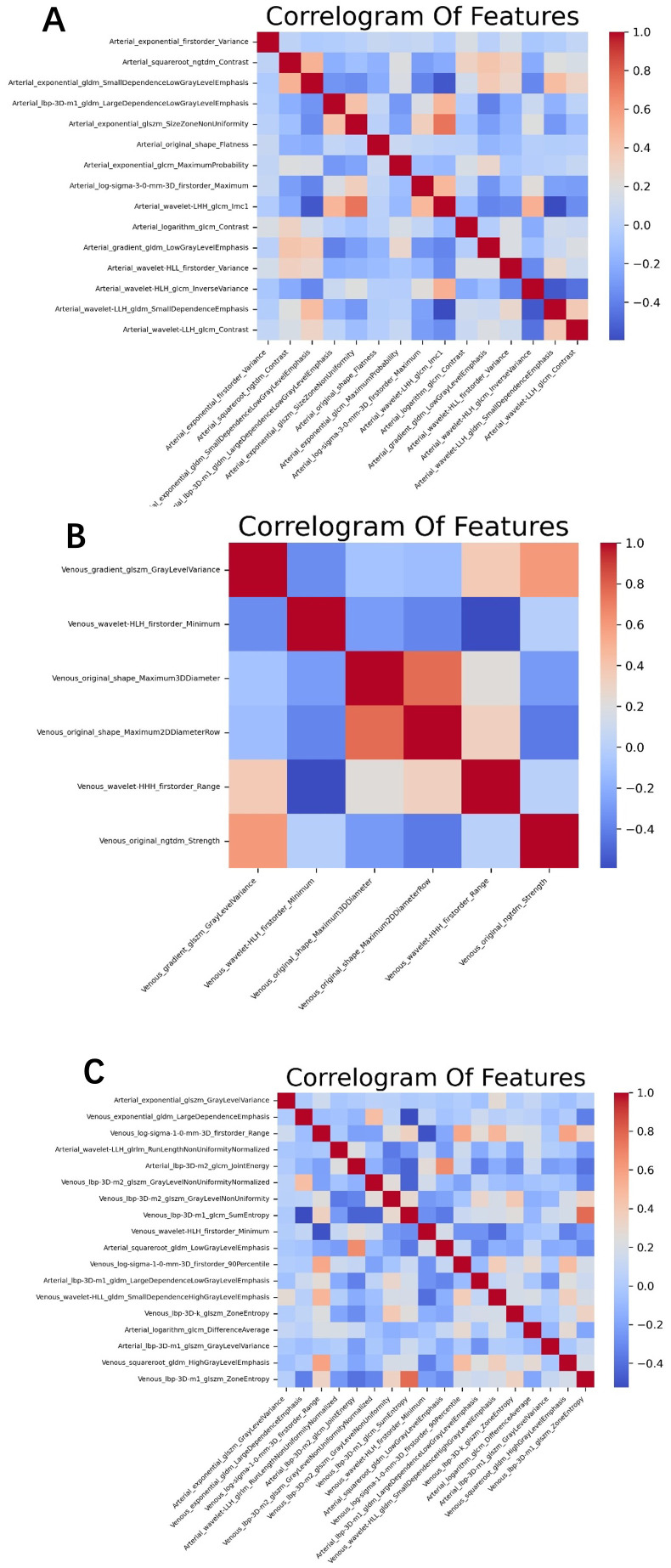
The optimal feature subset correlation heatmaps for **(A)** the arterial phase, **(B)** venous phase, and **(C)** the combined arterial and venous phase. The correlations among these optimal features are relatively weak and independent.

Although we extracted many features, not all of them had clinical significance for predicting adrenal masses. To identify the best set of distinguishing features, we introduced four methods for radiomic feature selection and dimensionality reduction. First, we calculated the maximum mutual information between features using MI (Mutual Information) and selected the top 500 features for both the arterial and venous phases. Next, we used MRMR (Minimum Redundancy Maximum Relevance) to filter out redundant and irrelevant features, resulting in 100 features for each phase. Then, we applied LASSO (Least Absolute Shrinkage and Selection Operator) for dimensionality reduction, yielding 19 features for the arterial phase and 6 for the venous phase. Finally, we removed one of each pair of features with Pearson correlation coefficients greater than 0.8, leaving 15 features for the arterial phase and 6 for the venous phase. For the combined arterial and venous phase data, we sequentially applied MI, MRMR, and LASSO, and selected 1,000, 100, and 22 features, respectively. After removing one feature from each pair with Pearson correlation coefficients greater than 0.8, 18 features left. See the heatmap (**Figure 2**) for details.

### 2.6 Statistical analysis

Statistical analyses were performed using Python 3.9 (https://www.python.org). The predictive efficiency was assessed using the AUC of the ROC curve. The LASSO algorithm and ROC curve generation were implemented using the “sklearn” package, whereas T-tests were conducted with the “scipy” package. A p-value less than 0.05 was considered to indicate a statistically significant difference.

## 3 Results

### 3.1 Patient baseline characteristics and contrast clearance rate

A total of 416 patients were enrolled in this study: 221 in the adenoma group (105 women [47.5%] and 116 men [52.5%]), aged 26 to 95 years (mean 60 [52, 69] years), with an average lesion size of 19.8 mm, relative washout rate (RPW) 0.3 (0.2, 0.4), and absolute washout rate (APW) 0.4 (0.2, 0.5); and 195 in the metastasis group (41 women [21%] and 154 men [79%]), aged 37 to 91 years (mean 69 [60, 73] years), with an average lesion size of 23.4 mm, RPW 0.1 (0, 0.1), and APW 0.1 (-0.1, 0.3). Patients were randomly divided into a training group (291 patients) and a validation group (125 patients). Significant differences were found in gender, age, lesion size, RPW, and APW between the two groups (P < 0.001). RPW and APW are calculated as follows: RPW = (CT values on venous phase − CT values on delayed phase) × 100% ÷ CT values on venous phase;APW = (CT values on venous phase − CT values on delayed phase) × 100%÷(CT values on venous phase − CT values on unenhanced phase). RPW and APW are important pharmacokinetic parameters that describe the clearance rate of drugs in the body (19). See **Table 1** for details.

**Table 1:** Patient baseline characteristics and contrast clearance rate.

Variables	Total (n = 416)	0 (n = 221)	1 (n = 195)	p
Age, Median (Q1,Q3)	65 (57, 71)	60 (52, 69)	69 (60, 73)	< 0.001
Gender, n (%)				< 0.001
Female	146 (35.1)	105 (47.5)	41 (21)	
Male	270 (64.9)	116 (52.5)	154 (79)	
Size, Median (Q1,Q3)	20.4 (16.4, 26.4)	19.8 (16.6, 23.7)	23.4 (16.1, 32.4)	< 0.001
RPW, Median (Q1,Q3)	0.2 (0, 0.4)	0.3 (0.2, 0.4)	0.1 (0, 0.1)	< 0.001
APW, Median (Q1,Q3)	0.3 (0, 0.4)	0.4 (0.2, 0.5)	0.1 (-0.1, 0.3)	< 0.001

### 3.2 Feature extraction and machine learning

**Figure 3:**
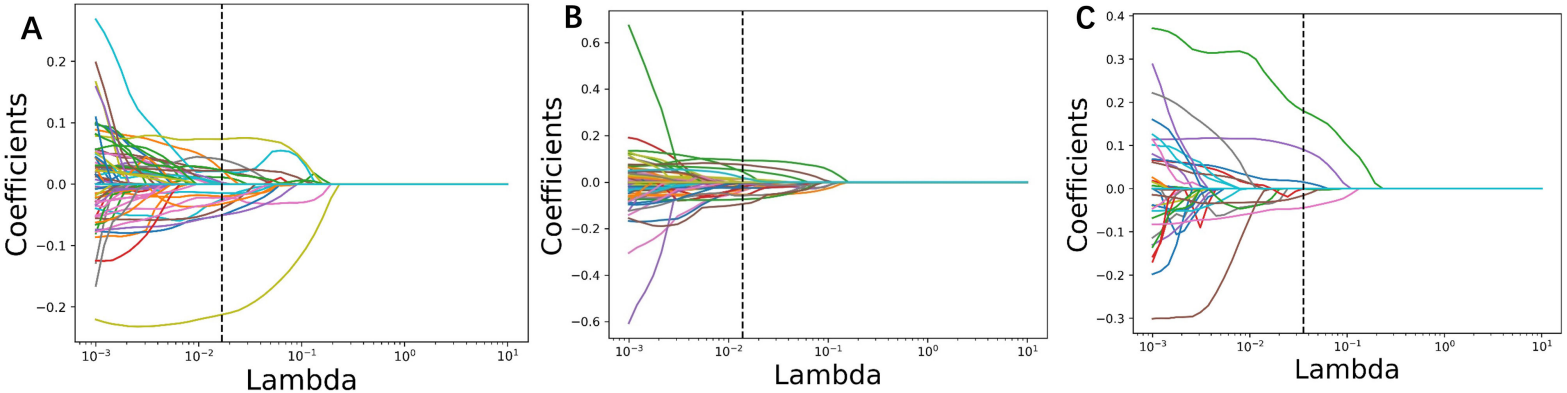
Radiomic parameters were screened using three steps: MI (Mutual Information), MRMR (Minimum Redundancy Maximum Relevance), and LASSO (Least Absolute Shrinkage and Selection Operator). The LASSO regression feature convergence plots for **(A)** the arterial phase, **(B)** venous phase, and **(C)** the combined arterial and venous phase data. Each curve represents the change in the coefficient of a particular feature.

In the training group comprising 291 patients, a total of 1,834 radiomic features were extracted from the arterial and venous phase images using 3D-Slicer. After feature selection through three steps—MI (Mutual Information), MRMR (Minimum Redundancy Maximum Relevance), and LASSO (Least Absolute Shrinkage and Selection Operator)—19 features were selected from the arterial phase, 6 from the venous phase, and 22 from the combined arterial and venous phase data (see **Figure 3** for the three respective images). Following Pearson correlation analysis, the optimal feature subsets for the three groups consisted of 15, 6, and 18 features, respectively. These features demonstrated the most robust performance in differentiating adrenal adenomas from metastases. The heatmap of the best features’ correlations (**Figure 2**, three respective images) revealed relatively weak and independent correlations among these features, indicating that all of them could be incorporated into the machine learning model.

**Figure 4:**
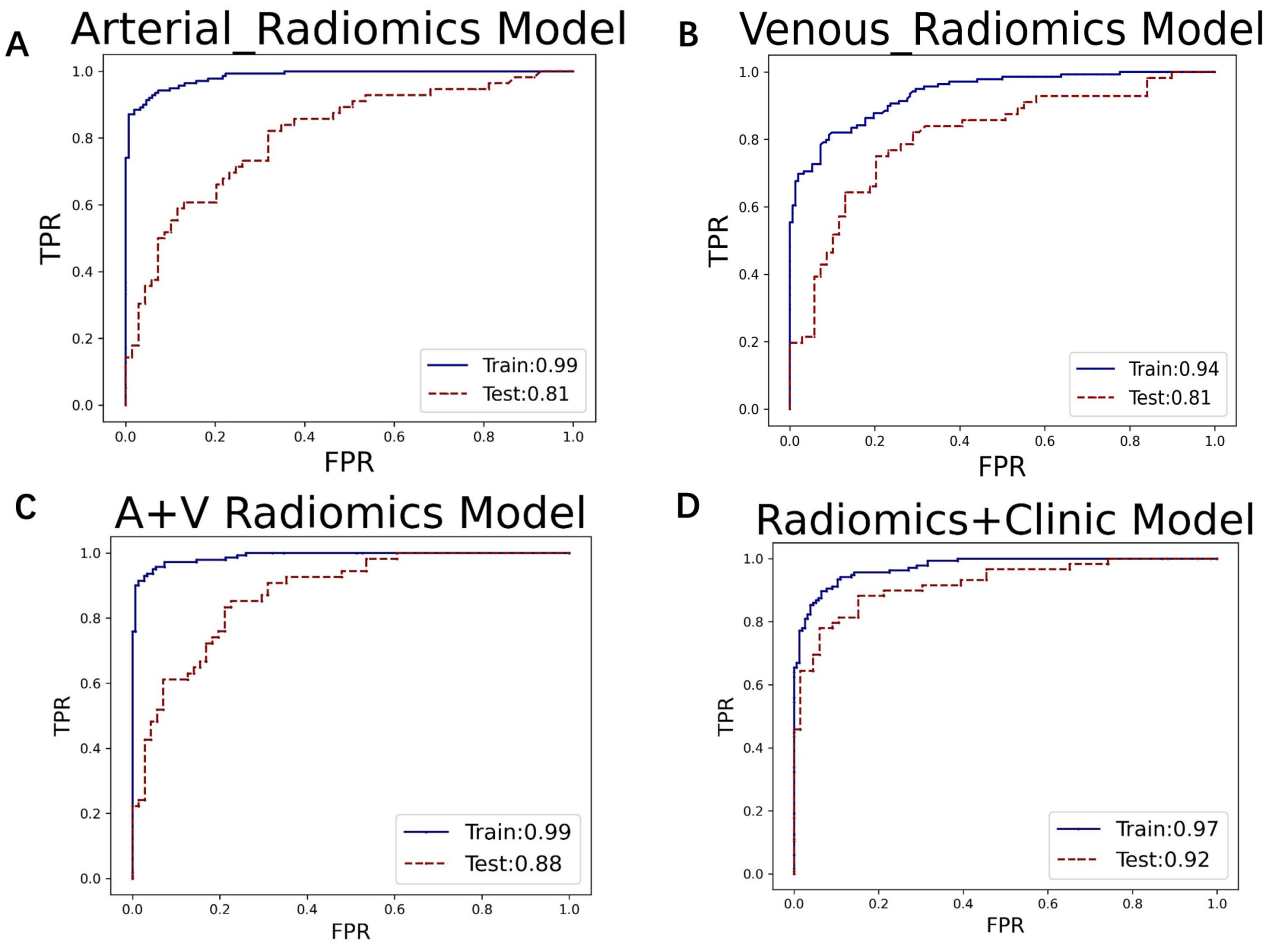
The ROC curves for **(A)** the arterial phase, **(B)** venous phase, **(C)** combined arterial and venous phase data, and **(D)** the integrated data of arterial and venous phases with clinical indicators in the XGBoost model. The AUC values for groups **(A–D)** are 0.81, 0.81, 0.88, and 0.92, respectively.

**Figure 5:**
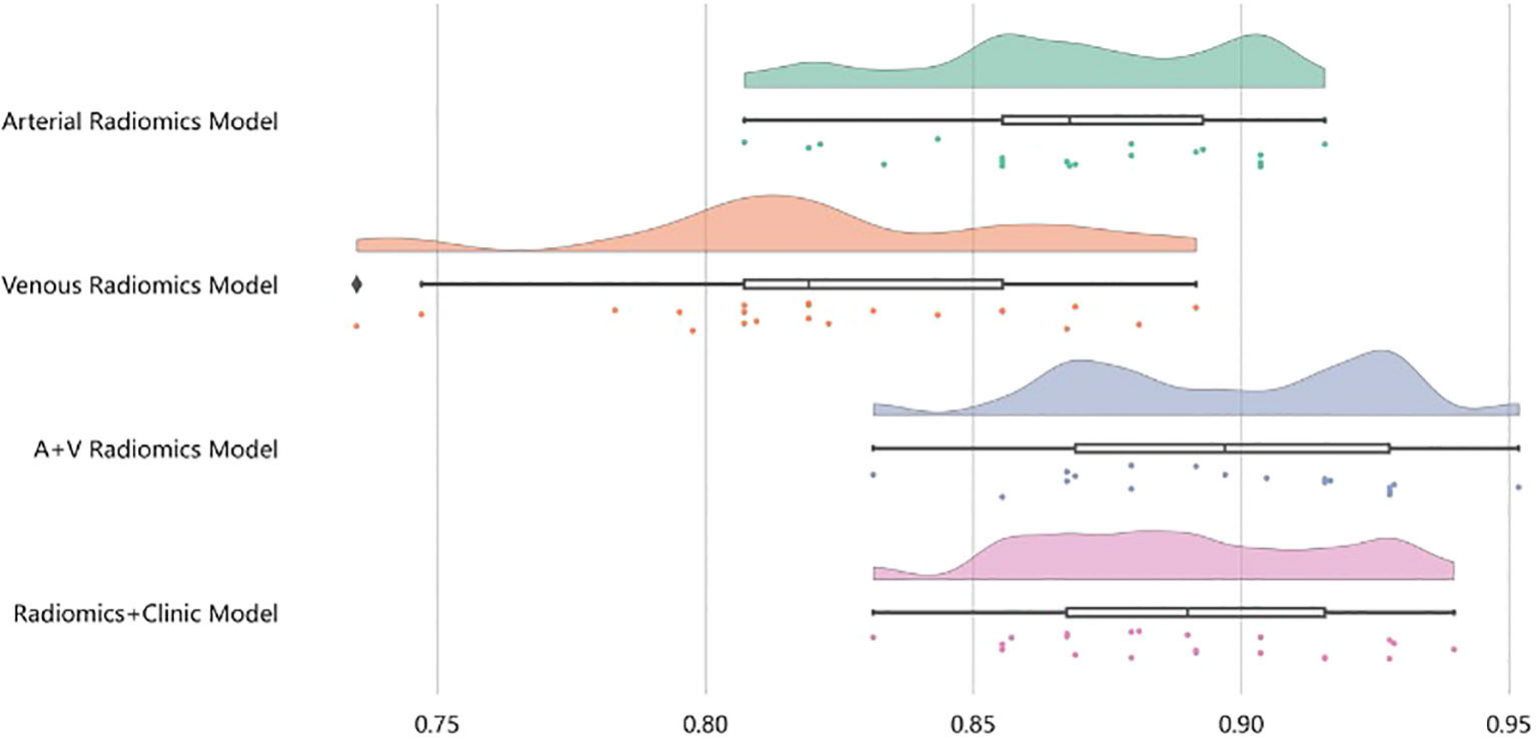
After four rounds of five-fold cross-validation, the results are shown in the figure. The average scores of the XGBoost model are 0.868, 0.823, 0.897, and 0.89, respectively.

**Figure 6:**
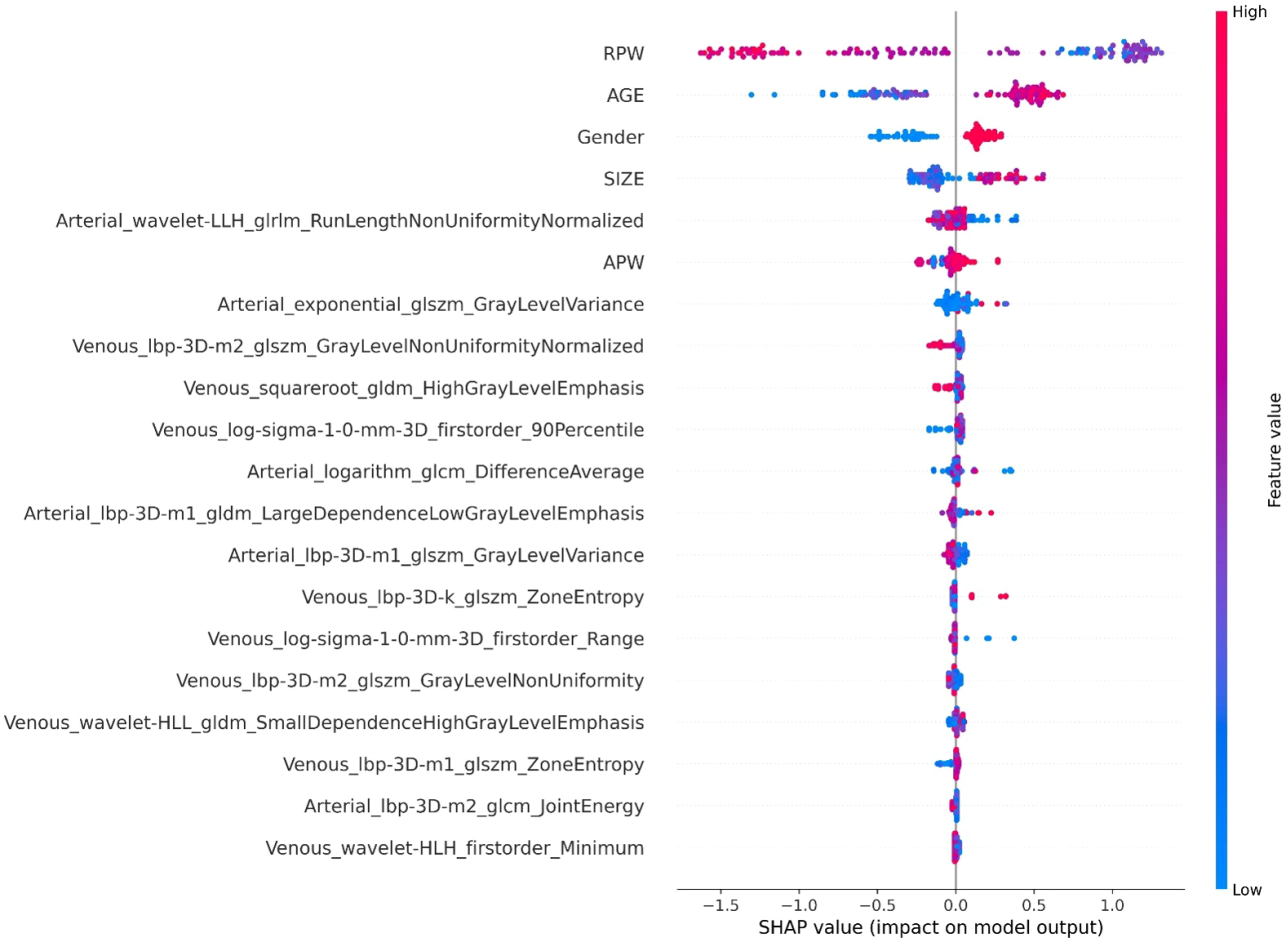
The SHAP Summary plot displays the SHAP values for each sample across different features.

**Figure 7:**
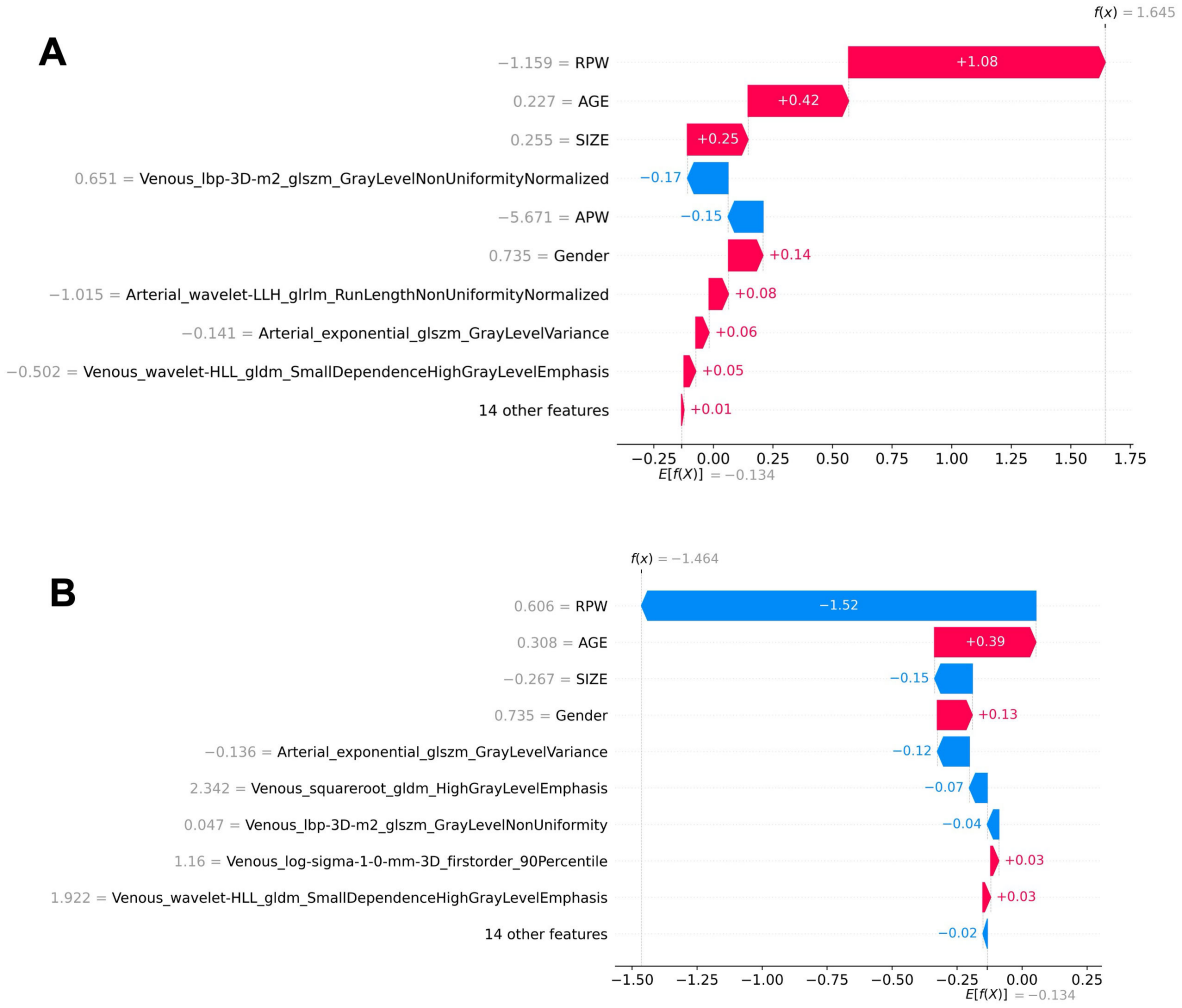
Local interpretability of individual prediction results using a waterfall plot. **(A)** The SHAP values for a positive case sample. **(B)** The SHAP values for a negative case sample. The plots illustrate the impact of each feature on the prediction outcome.

In the XGBoost model, the arterial phase AUC value, accuracy, precision, sensitivity, specificity, and F1 score were 0.81, 0.73, 0.70, 0.70, 0.75, and 0.70, respectively, with a 95% confidence interval of 0.73-0.89. The venous phase AUC value, accuracy, precision, sensitivity, specificity, and F1 score were 0.81, 0.75, 0.74, 0.70, 0.80, and 0.72, respectively, with a 95% confidence interval of 0.73-0.89. For the combined arterial and venous phase data, the AUC value, accuracy, precision, sensitivity, specificity, and F1 score were 0.88, 0.77, 0.77, 0.67, 0.85, and 0.71, respectively, with a 95% confidence interval of 0.81-0.94. For the combined arterial, venous, and clinical indicators data, the AUC value, accuracy, precision, sensitivity, specificity, and F1 score were 0.92, 0.83, 0.83, 0.81, 0.85, and 0.82, respectively, with a 95% confidence interval of 0.87-0.97. The results are shown in **Figure 4** and **Table 2**. After four rounds of five-fold cross-validation, the results are shown in **Figure 5**. The average scores of XGBoost were 0.868, 0.823, 0.897, and 0.89, respectively. The SHAP values for each sample under different features were plotted as a SHAP summary plot (**Figure 6**) to demonstrate the global interpretability of the model. Each point in the figure represents an observation. The x-coordinate of the point represents the SHAP value. On the y-axis, the ranking of the independent variables indicates the importance of the SHAP variables. That is, the importance of the independent variables decreases gradually from top to bottom. For example, RPW is the most influential feature, followed by AGE, Gender, and Size. Most of SHAP values for RPW is concentrated in the positive region, indicating that it has a positive impact on the model’s output. The color represents the magnitude of the feature value, with red indicating a high feature value and blue indicating a low feature value. In the RPW feature, red dots (high feature values) are concentrated in the negative SHAP value area, further confirming that high RPW values have a negative impact on the model’s output. Similarly, blue dots (low feature values) are concentrated in the positive SHAP value area, indicating that low RPW values have a positive impact on the model’s output. This interesting figure provides very important diagnosis information on adrenal adenomas and metastases, which cannot be realized based on traditional manual segmentation and diagnosis techniques. We can conclude that RPW, age and Gender are the three most important factors to be considered to differentiate lipid-poor adrenal adenomas from metastases. Besides, their feature values may imply different diagnosis outcomes, which provides clear guidelines for decision making on adrenal adenomas. While a high age and gender value indicates a positive impact on the model output, the RPW shows an opposite prediction, with high feature values implying a negative impact on the model output. **Figure 7** shows the local interpretability of individual prediction results with a Waterfall plot, with **Figure 7A** showing the SHAP values for a single positive case sample and **Figure 7B** showing the SHAP values for a single negative case sample, intuitively presenting the impact of each feature on the prediction results.

**Table 2:** The results of the XGBoost models.

	AUC	Accuracy	Precision	Sensitivity	Specificity	F1 Score	95%CI	RepeatedKFold
Arterial Radiomics Model	0.81	0.73	0.7	0.7	0.75	0.7	0.73-0.89	0.868
Venous Radiomics Model	0.81	0.75	0.74	0.7	0.8	0.72	0.73-0.89	0.823
A+V Radiomics Model	0.88	0.77	0.77	0.67	0.85	0.71	0.81-0.94	0.897
Radiomics+ClinicModel	0.92	0.83	0.83	0.81	0.85	0.82	0.87-0.97	0.89

## 4 Discussion

An adrenal incidentaloma is an adrenal mass discovered unintentionally during imaging studies conducted for reasons unrelated to suspected adrenal disease (20, 21). Their etiology and types are complex and diverse, and their prognoses vary widely. Therefore, it is crucial to differentiate benign and malignant lesions using imaging examinations and to manage adrenal diseases in a timely and appropriate manner (22). The 2016 and 2023 guidelines suggest that additional diagnostic work should only be performed for patients with adrenal masses ≥1 cm, who do not exhibit clinical symptoms or signs of excess adrenal hormones (7, 23). Adrenal adenomas and metastases, the most common benign and malignant adrenal tumors, respectively, are heterogeneous in traditional medical imaging (24). However, when adrenal adenomas develop with atypical signs and sizes, it becomes challenging to distinguish them from metastases (12). This study proposes a multi-modal machine learning model based on automatic segmentation techniques to predict adrenal adenomas and metastases. The application of automatic segmentation technology provides a solid foundation for radiomics analysis in the differential diagnosis of these two types of lesions. By using 3D Slicer and the large automatic segmentation model Medical SAM, we can quickly and accurately extract adrenal lesion areas from multi-modal enhanced CT images, significantly improving work efficiency and reducing human error. This technology enables more precise and efficient extraction of radiomics features, facilitating the identification of subtle lesion characteristics and providing a high-quality data foundation for subsequent machine learning model training (25). The multi-modal radiomics approach, including arterial phase, venous phase, combined arterial and venous phase data, and the fusion of clinical indicators with arterial and venous phase data, can fully utilize the rich information provided by different imaging stages combined with clinical features. Clinical data includes patient gender, age, lesion size, and imaging features (e.g., RPW and APW.), where RPW and APW refer to the absolute and relative clearance rates calculated using a 15-minute delayed scanning method, respectively. Reports have indicated that typical adrenal adenomas exhibit rapid washout characteristics, which can serve as a basis for differentiating adrenal adenomas from metastases (26). Although this method has high specificity and sensitivity, it is limited by long scan times, additional radiation, and contrast agents (27). In this study, images from different phases reflect the hemodynamic features of adrenal lesions at various time points, which are significant for distinguishing benign and malignant lesions. For example, adrenal adenomas usually have richer blood perfusion features because they are typically well-vascularized. In contrast, metastases, often originating from malignant tumors of other organs, have different vascularization and blood perfusion patterns from primary adrenal lesions. In imaging examinations, metastases may show uneven contrast agent distribution, reflecting their complex vascular structure and unstable blood perfusion. By integrating multi-modal radiomics features, the model can comprehensively capture lesion characteristics, thereby significantly improving diagnostic accuracy and enhancing the model’s discriminative ability.

In this study, the machine learning model played a crucial role in differentiating adrenal adenomas from metastases using clinical imaging data. Based on the multi-modal data obtained from automatic segmentation, the extracted radiomic features were classified using the XGBoost algorithm, which effectively enhanced the ability to differentiate adrenal adenomas from metastases. XGBoost, as a powerful ensemble learning algorithm, is capable of automatically handling the complex relationships between features and optimizing model performance through gradient boosting methods (28). The study results showed that the AUC values of the XGBoost model for the arterial phase, venous phase, combined arterial and venous phase data, and combined arterial, venous phase, and clinical indicators were 0.81, 0.81, 0.88, and 0.92, respectively, indicating a high diagnostic efficiency of the model. Additionally, through five-fold cross-validation, the average scores of the model were 0.868, 0.823, 0.897, and 0.89, respectively, which further demonstrated the stability and reliability of the model. The application of the machine learning model not only improved the accuracy of diagnosis but also provided strong support for clinical decision-making, which is critically important to reduce unnecessary invasive examinations and improve patient treatment outcomes and life quality. Regarding the interpretability of the machine learning model’s predictions, we conducted SHAP (SHapley Additive exPlanations) analysis. The SHAP values can intuitively show the contribution of each feature to the model’s predictive outcomes, thereby assisting clinicians in better understanding the model (29). In this study, the SHAP summary plot clearly indicated the impact of different features on the model and demonstrated that features such as RPW, age, gender, and lesion size had the most significant influence on model predictions. For example, lesions with higher RPW are more likely to be predicted as adrenal adenomas, which is consistent with clinical experience. Moreover, SHAP analysis can also provide local explanations for individual prediction results, which intuitively presents the impact of each feature on the predictive outcomes through Waterfall plots and further enhances the model’s interpretability and clinical applicability (30). This interpretative analysis not only helps to increase clinicians’ trust in machine learning models but also facilitates the application of these models in real clinical settings (31).

This article has some limitations: (1) Although the sample size is comparable to those in similar studies, it remains relatively limited for training and validating machine learning models, potentially compromising their generalization capability. (2) While five-fold cross-validation was employed in the study, the absence of external validation raises concerns about the reliability and generalizability of the findings. (3) Although SHAP analysis was utilized to explain the model’s predictions, the study offers only a limited discussion on feature selection and overall model interpretability. (4) While this study combines automatic segmentation and radiologist fine adjustments to realize accuracy considering the complexity of adrenal masses, the limited training case set may introduce potential biases in the automatic segmentation. A large data set and ML consistent iteration are expected to further improve model accuracy. (5) This study of this ML model is focused on CT radiomics but the incorporation of this model into other clinical imaging techniques (e.g., MRI or dual-energy CT) is still challenging due to the variations in imaging contrast technologies and mechanisms. Solving this limit is expected to provide synergistic diagnosis by combing different features of adrenal lesions and allow for developing generic ML models for imaging-based disease treatment and planning.

Multimodal enhanced CT radiomics, when combined with machine learning models based on automatic segmentation technology, hold significant potential for the differential diagnosis of adrenal adenomas and metastases. The use of automatic segmentation provides a high-quality data foundation essential for robust radiomics analysis (32). Multimodal radiomics can fully utilize information from different imaging stages, and machine learning models effectively improve the accuracy and reliability of diagnosis (33). SHAP analysis further enhances model interpretability, offering intuitive decision support for clinicians. Future research could benefit from larger sample sizes, external validation, and continued refinement of automatic segmentation techniques. Additionally, exploring a wider range of imaging modalities and feature extraction methods, as well as integrating machine learning models more seamlessly into clinical workflows, may contribute to more comprehensive and precise solutions for the diagnosis and treatment of adrenal tumors. To this end, refining the current model is a good starting point to extend this ML-based diagnosis technique to other imaging modalities, which is expected to provide insights in applying artificial intelligence to interpret clinical data for imaging and theranostics. The next approach to intelligent theranostics will be to develop deep learning, in addition to the supervised learning presented in this work, to automatic imaging processing and machine-based clinical decision making, providing more powerful and diverse ML models for imaging-based diagnosis on not only adrenal adenomas but also other diseases.

## Data Availability

The original contributions presented in the study are included in the article/**Supplementary Material**. Further inquiries can be directed to the corresponding authors.
